# How Kindergarten Teachers Adapt Their Speech for Children: Acoustic Evidence and Educational Implications from Mandarin Child-Directed Speech

**DOI:** 10.3390/bs16081330

**Published:** 2026-08-03

**Authors:** Zhehao Huang, Yishun Gu, Chenyu Hu, Xinlu Cai, Zhenqi Jiang, Qiang Guo, Minmin Yin

**Affiliations:** 1 Zhejiang Philosophy and Social Science Laboratory for Research in Early Development and Childcare, Hangzhou Normal University, Hangzhou 311121, China; huangzh@hznu.edu.cn (Z.H.); caixinlu@hznu.edu.cn (X.C.); 2 Jing Hengyi School of Education, Hangzhou Normal University, Hangzhou 311121, China; 2024112004143@stu.hznu.edu.cn (Y.G.); 2025112004206@stu.hznu.edu.cn (C.H.); 2020211909092@stu.hznu.edu.cn (Z.J.); 3 Chinese Education Modernization Research Institute, Hangzhou Normal University, Hangzhou 311121, China; 4 College of Child Development and Education, Zhejiang Normal University, Hangzhou 311231, China; guoq1989@zjnu.edu.cn

**Keywords:** child-directed speech (CDS), adult-directed speech (ADS), corner vowels, kindergarten teachers, fundamental frequency, vowel space area

## Abstract

Teachers play a central role in shaping children’s early language environments through the way they speak. Child-directed speech (CDS), characterized by distinct acoustic and prosodic adjustments, has been shown to facilitate children’s phonetic perception and language learning. This study investigates how Mandarin-speaking kindergarten teachers adapt their speech when addressing children compared to adults. Speech samples were collected from 35 female teachers using interactive puppet-based scenarios featuring Peppa Pig and Xiong Da. Acoustic analyses focused on the corner vowels /a/, /i/, and /u/, examining mean fundamental frequency (F_0_), F_0_ range, the first (F_1_) and second (F_2_) formant frequencies, and vowel space area (VSA). Results revealed that, relative to adult-directed speech (ADS), teachers’ CDS showed significantly longer vowel durations for /a/, /i/, and /u/ (all *p* < 0.001, surviving Bonferroni correction). In addition, descriptive trends—which did not survive Bonferroni correction—were observed for wider F_0_ range for /a/ (uncorrected *p* = 0.004), higher F_2_ for /a/ (uncorrected *p* = 0.011), and higher mean F_0_ for /u/ (uncorrected *p* = 0.002); these effects should be interpreted with caution and are not considered statistically significant in the present study. Vowel space area did not differ significantly between CDS and ADS. These findings indicate systematic adaptations in prosodic and temporal features—particularly duration and pitch variability—though not all acoustic measures showed significant changes. Beyond acoustic distinctions, the results underscore teachers’ active role in modulating their speech to support early language development. The study contributes to understanding how Mandarin-speaking kindergarten teachers acoustically adapt their speech to children and offers preliminary insights that may inform future research on teacher speech-training and early-childhood communication practices.

## 1. Introduction

When addressing young children, adults and older children adapt their speech and use a specialized register known as child-directed speech (Ko & Jun, 2024). CDS is marked by higher and more variable pitch, simplified vocabulary, shorter utterances, and changes in articulatory and phonetic properties. It has been shown to support early language development (Soderstrom, 2021). The hyperarticulation and pragmatic hypotheses propose that CDS facilitates acquisition by increasing phonetic contrasts, capturing children’s attention, and supporting social interaction (Cristia & Seidl, 2014).

While substantial research has documented CDS in parent–child dyads (Porritt et al., 2014; Newman et al., 2016), the acoustic properties of teacher CDS in classroom settings—particularly in tonal languages such as Mandarin—remain less well understood. This matters because kindergarten teachers provide a substantial share of children’s daily linguistic input and because pitch variations in tonal languages may interact with the prosodic features that are typically modulated in CDS.

To address these gaps, we investigate acoustic differences between CDS and adult-directed speech (ADS) produced by native Mandarin-speaking kindergarten teachers. Using a within-subjects design, we analyze the corner vowels /a/, /i/, and /u/ and measure fundamental frequency (F_0_), pitch range (F_0_ range), the first formant (F_1_), the second formant (F_2_), and vowel space area (VSA). By identifying systematic differences between CDS and ADS, this study provides empirical evidence of teacher speech adaptations and their potential implications for early language acquisition in Mandarin-speaking preschool children.

### 1.1. Theoretical Framework and Acoustic Characteristics of CDS

Child-directed speech (CDS) is a specialized speech register that adults use when addressing young children. It is characterized by higher and more variable pitch, slower speech rate, longer vowel durations, and more exaggerated articulatory patterns compared to adult-directed speech (ADS) (Ko & Jun, 2024; Soderstrom, 2021).

Two complementary theoretical accounts have been proposed to explain why speakers modulate their speech in this way. The Hyperarticulation Hypothesis (Cristia & Seidl, 2014) posits that speakers enhance phonetic contrasts—such as expanding vowel space and lengthening vowels—to facilitate phonological category learning in infants (Liu et al., 2003; Song et al., 2010). The Pragmatic/Functional Hypothesis (e.g., Burnham et al., 2002; Fernald & Hurtado, 2006; Nencheva et al., 2021) emphasizes the social and attentional functions of CDS: higher and more variable pitch captures children’s attention and conveys affective engagement, thereby supporting interactive learning (Kaplan et al., 1999; Nencheva et al., 2021).

These frameworks generate specific, testable predictions about acoustic modulation in CDS. Fundamental frequency (F_0_) and F_0_ range reflect pitch modulation, which is predicted to be higher and more variable in CDS under the Pragmatic Hypothesis. Vowel duration is a key index of temporal clarity, predicted to be longer in CDS under the Hyperarticulation Hypothesis. Formant frequencies (F_1_ and F_2_) and the derived vowel space area (VSA) directly index articulatory hyperarticulation: a larger VSA indicates more distinct vowel targets, predicted to facilitate phonetic category learning (Liu et al., 2003). These measures are particularly relevant for corner vowels (/a/, /i/, /u/), which define the boundaries of the vowel space and serve as common reference points in developmental phonetic research (Sarvasy et al., 2022; Steen & Englund, 2022). The vowel space area derived from these three vowels is a well-validated proxy for overall articulatory hyperarticulation (Liu et al., 2003), and using this established set of vowels ensures comparability with prior CDS studies across languages. While Mandarin has additional vowels and diphthongs (e.g., /ə/, /ɤ/, /y/, /ai/, /ei/), the corner vowels provide the most parsimonious and reliable index of global vowel space expansion—the primary question of interest in the present study.

While extensive research has documented these acoustic features in parent–child dyads (e.g., Porritt et al., 2014; Newman et al., 2016), much less is known about CDS in educational settings or in tonal languages such as Mandarin. This gap matters for two reasons. First, for many preschoolers, kindergarten teachers provide a substantial share of daily linguistic input, yet teacher speech in classrooms remains understudied (Steen & Englund, 2022). Second, in tonal languages, where pitch encodes lexical contrasts, prosodic adjustments in CDS may interact with phonological processing in ways that differ from non-tonal languages. These considerations directly motivate the present acoustic analysis of Mandarin-speaking kindergarten teachers’ CDS.

### 1.2. Corner Vowel Features and Hyperarticulation

Among the acoustic features that differentiate CDS from ADS, the corner vowels /a/, /i/, and /u/ have drawn particular attention. They mark the outer limits of the vowel space and serve as reference points for assessing articulatory hyperarticulation (Sarvasy et al., 2022; Steen & Englund, 2022). In CDS, these vowels are often produced with more extreme formant values, longer durations, and higher F_0_, reflecting the broader acoustic enhancements predicted by the Hyperarticulation Hypothesis (Liu et al., 2003; Cristia & Seidl, 2014).

The vowel space area (VSA)—defined by plotting F_1_ and F_2_ values in a two-dimensional space—is a key metric for assessing vowel distinctiveness. A larger VSA is associated with increased phonetic clarity and has been linked to better vowel discrimination in infants (Liu et al., 2003). However, most evidence for VSA expansion comes from infant-directed speech; studies on preschool-aged children are limited, and findings from Norwegian kindergartens suggest that VSA may not be expanded in teacher CDS addressed to older children (Steen & Englund, 2022).

We acknowledge that Mandarin has a richer vowel inventory beyond the three corner vowels, including mid vowels (/ə/, /ɤ/), front rounded vowels (/y/), and various diphthongs (e.g., /ai/, /ei/, /ou/, /uo/), all of which are relevant to child language acquisition. However, the primary objective of this study was to assess global vowel space expansion as an index of articulatory hyperarticulation—a construct most effectively captured by the extreme positions of the vowel space (corner vowels). Focusing on the corner vowels also allows for direct comparability with foundational CDS studies (Liu et al., 2003; Sarvasy et al., 2022; Steen & Englund, 2022). Investigating the full range of Mandarin vowels and diphthongs would be a valuable direction for future research.

This gap—whether Mandarin-speaking kindergarten teachers hyperarticulate vowels when addressing preschoolers—motivates the present investigation of F_0_, F_0_ range, duration, F_1_, F_2_, and VSA for the corner vowels /a/, /i/, and /u/.

### 1.3. Preschool Language Environment and Teacher Speech

Caregivers are traditionally viewed as the main source of linguistic input in early development. For many preschoolers, however, teachers now play a central role in the daily language environment. Building on recent work on teacher CDS in Norwegian kindergartens (Steen & Englund, 2022), the present study extends this line of inquiry to Mandarin-speaking classrooms, where the tonal nature of the language offers new theoretical insights.

Preschool environments provide abundant peer and adult interactions that shape language growth. Lekhal et al. (2011) reported that children who entered center-based care at 18 months showed lower rates of late talking at age three than children cared for only at home. Consistent with this, Steen and Englund (2022) showed that Norwegian kindergarten teachers produce CDS with higher pitch, wider pitch range, and longer vowel durations. These features are known to facilitate language learning.

These results support the view of CDS as a flexible register that adults, including teachers, adjust to meet young listeners’ needs. Yet most research centers on parent–child interaction. Teacher speech in classrooms remains understudied, especially in tonal languages such as Mandarin.

In Mandarin-speaking settings, scholars emphasize training kindergarten teachers to recognize and apply CDS strategies; documenting how teachers adjust their speech has both theoretical value and practical implications for curriculum design, teacher training, and language intervention. Despite these considerations, empirical studies of Mandarin CDS in preschool classrooms remain scarce, especially on acoustic detail. This gap is striking because Mandarin is tonal. Pitch encodes lexical contrasts and may interact with the prosodic adjustments typical of CDS.

The present study examines the acoustic properties of CDS produced by Mandarin-speaking kindergarten teachers by comparing it with their ADS, with the goal of advancing cross-linguistic accounts of CDS.

### 1.4. The Present Contribution

While prior research has examined teacher CDS in Norwegian kindergartens (Steen & Englund, 2022) and Mandarin CDS in parent–child dyads (Liu et al., 2003; Liu et al., 2009), the present study advances existing knowledge in three specific ways. First, it provides the first systematic acoustic analysis of all three corner vowels (/a/, /i/, /u/) across multiple acoustic parameters (F_0_, F_0_ range, duration, F_1_, F_2_, VSA) in Mandarin-speaking kindergarten teachers, using a within-subjects design that controls for speaker-specific variation. Second, it directly tests competing predictions from the Hyperarticulation Hypothesis (which predicts vowel space expansion) and the Pragmatic/Functional Hypothesis (which emphasizes prosodic modulation such as pitch and duration), contributing to theoretical debates about which acoustic features are most consistently modulated in CDS and under what developmental conditions. Third, by examining CDS directed at preschoolers (3–6 years) rather than infants, it extends the developmental scope of CDS research and provides evidence for age-sensitive modulation—testing whether the hyperarticulation patterns documented in infant-directed speech are maintained, reduced, or replaced by other acoustic enhancements when children are older.

### 1.5. The Present Study: Research Questions and Hypotheses

The preceding review has highlighted two main gaps in the existing literature: first, the scarcity of acoustic studies on CDS in non-familial educational settings; and second, the limited evidence on CDS in tonal languages such as Mandarin, where pitch variations carry lexical meaning. To address these gaps, the present study investigates the acoustic properties of corner vowels (/a/, /i/, /u/) produced by Mandarin-speaking kindergarten teachers in CDS compared to their ADS.

Specifically, we address the following research questions: RQ1: Do Mandarin-speaking kindergarten teachers systematically modify the acoustic properties of corner vowels when addressing children versus adults? RQ2: Which acoustic parameters (F_0_, F_0_ range, duration, F_1_, F_2_, VSA) show significant adaptations in CDS, and do these adaptations vary across vowels? RQ3: Is there evidence of vowel space expansion in teacher CDS, consistent with the hyperarticulation hypothesis?

Based on prior cross-linguistic findings on CDS, we formulated the following hypotheses: H1 (Pitch): Teachers will produce CDS with higher mean F_0_ and wider F_0_ range than ADS, as elevated and more variable pitch is thought to attract children’s attention and convey affective engagement (Burnham et al., 2002; Kaplan et al., 1999; Liu et al., 2003). H2 (Duration): Teachers will produce CDS with longer vowel durations than ADS, reflecting slower speech rate and increased temporal clarity, which facilitates word segmentation and lexical learning (Song et al., 2010; Zangl et al., 2005). H3 (Formants): Teachers will produce CDS with higher F_1_ and F_2_ values than ADS, reflecting more hyperarticulated (i.e., more extreme) vowel targets, consistent with the hyperarticulation hypothesis (Cristia & Seidl, 2014; Liu et al., 2003). H4 (VSA): Teachers will produce CDS with a larger vowel space area (VSA) than ADS, as expanded vowel space is a key index of articulatory hyperarticulation and has been associated with enhanced phonetic clarity (Liu et al., 2003; Sarvasy et al., 2022; Steen & Englund, 2022). H5 (Vowel-specificity): Given that different vowels may be differentially affected by speech style adaptations (e.g., due to articulatory constraints or lexical frequency), we treat the pattern of adaptations across /a/, /i/, and /u/ as exploratory rather than making specific directional predictions for each vowel.

We note that while the hyperarticulation hypothesis predicts VSA expansion in CDS, recent findings from Norwegian kindergarten teachers (Steen & Englund, 2022) have shown reduced VSA in CDS addressed to preschoolers (aged 3–6 years), suggesting that VSA expansion may be modulated by the age of the child listener. Given that our child participants were in the same age range (3–6 years), we also consider the possibility that VSA may not be expanded, or may even be reduced, in this age group. We return to this point in the Discussion.

## 2. Materials and Methods

### 2.1. Participants

This study included two categories of participants: preschool teachers and preschool-aged children. The present section outlines the inclusion criteria and demographic information for the teacher sample.

#### 2.1.1. Kindergarten Teachers

Given that acoustic features of CDS may vary with speaker gender and native language and that the majority of kindergarten teachers in China are female native speakers of Mandarin, only female teachers who identified Mandarin as their first language were recruited for this study. This sampling decision reflects the typical linguistic environment of Chinese preschool classrooms. However, we acknowledge that this gender-restricted sample limits the generalizability of our findings to male teachers or to educational contexts with different gender distributions.

A total of 35 female kindergarten teachers were recruited from multiple kindergartens in Hangzhou, China. Participants ranged in age from 23 to 43 years (M = 30.6, SD = 5.49). Their teaching experience varied from 1.5 to 36 years (M = 13.03, SD = 10.96). In terms of educational background, 88.57% held a bachelor’s degree in early-childhood education, while 11.43% held an associate’s degree, as shown in Table 1.

Inclusion Criteria for Teachers:

- Native speakers of Mandarin Chinese;
- In good physical health at the time of data collection, with no reported symptoms of cold, sore throat, or related voice disorders.

##### Sample Size Justification and Power Analysis

No a priori power analysis was conducted prior to data collection, as this study was exploratory in nature and, at the time of design, no prior studies had examined the acoustic properties of Mandarin-speaking kindergarten teachers’ CDS with a within-subjects design from which effect sizes could be estimated. However, we conducted a post hoc sensitivity power analysis to determine the minimum effect size detectable with our sample of 35 teachers. For a within-subjects repeated-measures design with two conditions (CDS vs. ADS), α = 0.05, power = 0.80, and N = 35, the minimum detectable effect size is Cohen’s f = 0.30 (equivalent to ηp^2^ ≈ 0.08), corresponding to a medium-to-large effect according to Cohen’s (1988) conventions. This indicates that our study is adequately powered to detect large acoustic differences between CDS and ADS but is underpowered to detect small-to-medium effects (ηp^2^ < 0.06). As shown in Section 3, several of our significant effects (e.g., duration for /a/, ηp^2^ = 0.723) substantially exceed this threshold, while nonsignificant findings (e.g., VSA, ηp^2^ = 0.016) are well below it. We acknowledge this limitation in interpreting our null findings and encourage replication with larger samples in future research.

#### 2.1.2. Preschool-Aged Children

A total of 35 preschool-aged children were recruited from the same classrooms as the 35 participating kindergarten teachers in Hangzhou, China. All children were enrolled in the teachers’ respective classes, ensuring that each teacher–child pair was familiar with one another through daily classroom interactions. This familiarity was intended to enhance the ecological validity of the CDS recordings by approximating natural teacher–child communication.

Role of child participants. Children were recruited exclusively to serve as interactive partners to elicit CDS from their teachers. They were not tested, assessed, or measured on any language, cognitive, or developmental outcomes. The acoustic analyses reported in this study focus exclusively on teachers’ speech production (CDS vs. ADS). No analyses linking child characteristics (age, gender, or language proficiency) to teacher speech adaptations were conducted as primary analyses. However, exploratory analyses examining whether child age modulated teacher acoustic adaptations are reported in Section 3.7.

Rationale for reporting child demographics. Child demographic information (age, gender) is reported solely to provide transparency about the characteristics of the interaction partners and to help readers contextualize the CDS samples. This is standard practice in CDS research (e.g., Steen & Englund, 2022; Liu et al., 2003) and facilitates cross-study comparability by allowing readers to evaluate the generalizability of findings to different child age groups. We acknowledge that child characteristics could plausibly influence teacher speech (e.g., teachers may adapt their speech differently to older vs. younger children), and we have addressed this possibility through exploratory analyses reported below.

Participants ranged in age from 3.7 to 6.6 years, with a mean age of 4.9 years (M = 4.9, SD = 1.0). The sample included 20 girls and 15 boys. Girls ranged in age from 3.9 to 6.6 years (M = 4.8, SD = 1.0), while boys ranged from 3.7 to 6.6 years, with a mean age of 5.02 years (M = 5.0, SD = 1.2), as shown in Table 2.

Inclusion Criteria for Preschool Children:

- Age between 3 and 6 years;
- Mandarin Chinese as their first language;
- No reported physical or psychological illness during the testing period, including colds or related health conditions.

#### 2.1.3. Ethical Approval and Informed Consent

This study was conducted in accordance with the Declaration of Helsinki. Ethical approval was obtained from the Human Experiment Ethics Committee of Zhejiang Normal University (protocol code ZSR72024028, approved on 10 March 2024). Written informed consent was obtained from all participating teachers and from the parents or legal guardians of all participating children prior to data collection. Participants were informed that their data would be used for research purposes only and that they could withdraw from the study at any time without consequence.

### 2.2. Experimental Procedure

All speech materials were drawn from semi-structured, elicited interactions between the participating teachers and the preschool-aged children in their own classrooms, conducted in familiar rooms within each kindergarten to ensure ecological validity and to minimize speaker discomfort. While the recordings took place in familiar classroom settings with familiar teacher–child pairs, the interactions followed a controlled elicitation protocol to ensure comparability of the target vowel sounds across participants and conditions. We therefore characterize the speech samples as semi-structured elicited speech rather than fully naturalistic spontaneous speech. This approach balances experimental control (ensuring cross-participant comparability of the target vowels /a/, /i/, and /u/) with ecological validity (using familiar settings and interaction partners).

All speech samples were captured with a digital recorder and monitored in Sound Forge 9.0 at 44.1 kHz and 16-bit resolution. Recordings took place in quiet rooms with ambient noise kept below 40 dB SPL. The microphone was placed 8 to 12 cm from the teacher’s mouth at an angle of approximately 45 degrees to reduce breathing noise. Teachers used child-familiar rooms, such as resource rooms (about 20 m^2^) or small reading rooms (about 9 m^2^), depending on availability.

To ensure recording quality, only the teacher, the child, and the experimenter were present in the room during each session. Noise-producing objects, such as musical instruments or electronic toys, were not allowed, and doors and windows remained closed. In most cases, CDS and ADS were recorded in the same room to control for room acoustics.

Each teacher participated in both CDS and ADS recording sessions. In the CDS condition, each teacher interacted with one child from her own classroom, with whom she had an established daily relationship. The child participated only in the CDS condition and was not present during the ADS session. The ADS condition involved a semi-structured conversation between the teacher and the adult experimenter, without any child listener present. This design allowed us to obtain a clear baseline of each teacher’s adult-directed speech under comparable phonetic content but without the influence of a child audience.

Role of child partners in the CDS condition. In the CDS condition, children were present exclusively as conversational partners to elicit naturalistic teacher speech. They were not instructed to perform any specific task or to respond in any particular way; rather, teachers were asked to interact with the child as they normally would in the classroom, using the toys as conversational prompts. The children’s primary function was to create a communicative context that would approximate real classroom interactions, thereby enhancing the ecological validity of the CDS recordings. Children were not present during the ADS condition, ensuring that any differences between CDS and ADS could be attributed to the presence of a child listener rather than to other task-related factors.

Prior to recording, the teacher was provided with a standardized instruction script explaining the purpose of the study:

“Hello, this study records your speech as a kindergarten teacher to examine how you talk to children and how you talk to adults. The data are for research use only and not for commercial purposes. Please speak as you normally do in daily interactions. You will use two toys, Peppa Pig and Xiong Da, to converse naturally with a child, for example: ‘Look, what is this?’ ‘This is Peppa Pig.’ ‘And what is this?’ ‘This is Xiong Da.’ After the interaction, the experimenter will ask a simple question, such as: ‘Which two toys did you use during your conversation with the child?’”

For CDS, teachers interacted with a child using two plush toys: Peppa Pig (小 (xiǎo)猪 (zhū) 佩 (pèi) 奇 (qí)) and Xiong Da (熊 (xióng) 大 (dà)). These items were chosen to elicit the corner vowels /a/, /i/, and /u/ in natural discourse through a controlled naming and conversation task. The use of two specific toys served to standardize the lexical content across teachers while still allowing for spontaneous conversational exchanges. For context, Steen and Englund (2022) instructed Norwegian kindergarten teachers to use six toys to elicit target vowels /a/, /i/, /u/, /a:/, /i:/, and /u:/ through natural dialogue: plush cake (ka:ke), plush kitten (kat:), plush tiger (ti:ger), Pippi Longstocking doll (pip:i), a touch-and-feel book (bu:k), and a Billy Goat doll (buk:) (Steen & Englund, 2022).

For ADS, each teacher held a semi-structured conversation with the same experimenter who facilitated the CDS session. During this phase, the experimenter prompted the teacher to talk about their recent activities with children and encouraged them to mention the names of the same two toys. This ensured the phonetic comparability of CDS and ADS samples, particularly with regard to the targeted vowel sounds.

Each recording session lasted between 1 and 5 min; CDS sessions averaged 3 min and ADS sessions average 2 min. Teachers were instructed to speak at their normal conversational pace and volume.

Importantly, while the recordings were elicited under controlled task conditions to ensure cross-participant comparability, they took place in familiar school environments with familiar interaction partners, which we believe provides a reasonable balance between experimental control and ecological validity.

#### Speech Material Quantity

In total, approximately 105 min of CDS (mean = 3.0 min per teacher, range = 1–5 min) and 70 min of ADS (mean = 2.0 min per teacher, range = 1–5 min) were recorded and analyzed. The longer CDS sessions reflect the interactive nature of teacher–child conversations, which included turn-taking, questioning, and naming of toys, whereas ADS sessions were more direct and concise. Despite the difference in session duration, the number of analyzable vowel tokens per condition was balanced (see Section 2.4, Selection and Exclusion of Vowel Tokens), as the ADS sessions yielded sufficient tokens due to the higher density of target vowels in the elicited naming responses.

### 2.3. Experimental Design

This study used a within-subjects (repeated-measures) design to compare the acoustic properties of the corner vowels /a/, /i/, and /u/ in Mandarin-speaking kindergarten teachers’ CDS and their ADS. Audio recordings were obtained from 35 female preschool teachers who were native speakers of Mandarin, yielding 70 speech samples across the two conditions (35 CDS + 35 ADS).

The independent variable was Speech Type (CDS vs. ADS), manipulated within each participant. The dependent variables were the acoustic parameters of the three target corner vowels (/a/, /i/, /u/), including: mean fundamental frequency, F_0_ range, vowel duration, first formant, second formant, and vowel space area. For each vowel and each acoustic parameter, separate analyses were conducted to evaluate the effect of Speech Type.

### 2.4. Data Processing

This study examined the corner vowels /a/, /i/, and /u/ in both CDS and ADS. Speech materials were captured with a digital recorder and then analyzed using the Dr. Speech Science for Windows software package (Version 4.0, Tiger Electronics, Seattle, WA, USA), running on a Dell Precision workstation with 16 GB RAM. For each vowel token, we measured the following acoustic parameters: F_0_, F_0_ range, vowel duration, F_1_, F_2_, and VSA.

F_0_, F_0_ range, vowel duration, and VSA were calculated based on F_1_ and F_2_ values. Vowel duration was measured from the onset to the offset of the vowel. The F_0_ range for each target vowel was calculated by subtracting the minimum F_0_ from the maximum F_0_, where both values were measured across the entire voiced duration of that vowel. This method captures the full extent of pitch modulation within each vowel and is sensitive to the dynamic prosodic exaggerations typical of child-directed speech. All acoustic parameters were measured in Hertz (Hz), except for vowel duration, which was measured in seconds (s).

#### 2.4.1. Acoustic Analysis Settings and Pre-Processing

Prior to formant extraction, all speech samples were pre-processed using a high-pass filter with an 80 Hz cutoff frequency to remove low-frequency ambient noise and microphone rumble. A pre-emphasis factor of 0.95 was applied to enhance high-frequency spectral information and improve formant tracking reliability, consistent with standard acoustic analysis protocols (Ladefoged & Johnson, 2015). All analyses were performed on the full-bandwidth signals (44.1 kHz sampling rate, 16-bit resolution).

For formant extraction, Linear Predictive Coding (LPC) was used with the following settings: LPC order of 14 for female voices (following the recommendations of the DRS system manual and prior studies, e.g., Liu et al., 2003), Hamming window with window length of 25 ms and frame shift of 10 ms. The maximum formant frequency was set to 6000 Hz for female speakers, and the number of formants extracted was set to 4, consistent with standard practice for adult female voices (Vorperian & Kent, 2007). Pre-emphasis was applied prior to LPC analysis with a factor of 0.95, as specified above. These settings were chosen based on the DRS system manual recommendations and validated through pilot testing to ensure reliable formant tracking across all vowel categories.

#### 2.4.2. Segmentation and Formant Extraction

Vowel onset and offset were manually marked with reference to LPC spectra, wideband spectrograms (300 Hz analysis bandwidth), and waveform displays, following standard procedures in phonetic analysis (Ladefoged & Johnson, 2015). Vowel duration was defined as the interval from onset to offset. F_1_ and F_2_ were sampled at the temporal midpoint (50% of vowel duration) of each vowel token to minimize coarticulatory effects from adjacent consonants (Steen & Englund, 2022). For each token, the LPC-generated formant trajectories were visually overlaid on the wideband spectrogram to confirm tracking accuracy before accepting the midpoint values.

No frequency normalization (e.g., z-score normalization, Lobanov normalization, or speaker-intrinsic normalization) was applied to formant values. This decision was made for three reasons. First, all comparisons in this study are within-subjects (each teacher’s CDS compared to her own ADS), which inherently controls for speaker-specific anatomical differences in vocal tract length and shape. Second, normalization procedures can inadvertently remove meaningful between-speaker variability that reflects genuine differences in speech production. Third, this approach aligns with prior CDS studies that have employed similar within-subjects designs and did not apply normalization (e.g., Steen & Englund, 2022; Liu et al., 2003; Sarvasy et al., 2022), facilitating direct cross-study comparability. We acknowledge that normalization may be beneficial in cross-speaker comparisons; however, given the within-subjects design of the present study, we consider it appropriate to report raw formant values.

#### 2.4.3. Selection and Exclusion of Vowel Tokens

A total of 35 teachers participated in the study, and from each teacher we analyzed both CDS and ADS recordings. For each recording, we initially segmented all instances of the target corner vowels /a/, /i/, and /u/ that occurred in lexical items produced during the semi-structured interactions. Vowel tokens were included only if they satisfied the following criteria: (a) the vowel was produced with a clear, noise-free signal, with no overlapping speech from the child or experimenter; (b) the vowel was not produced with excessive background noise (ambient noise consistently < 40 dB SPL); (c) the vowel was not produced with vocal fry or creaky voice that prevented reliable formant tracking; and (d) F_1_ and F_2_ were clearly visible on the wideband spectrogram and could be reliably estimated by the LPC algorithm.

On average, approximately 13 tokens per vowel per condition per speaker were successfully segmented and retained for analysis, yielding a total of 2730 vowel tokens (1365 in CDS and 1365 in ADS). Across conditions and vowels, the number of retained tokens per speaker ranged from 10 to 16 per vowel per condition. Token counts did not differ substantially across vowels or between CDS and ADS; the average token counts per vowel were as follows: /a/ = 13.4 (SD = 1.1), /i/ = 12.8 (SD = 1.3), /u/ = 13.1 (SD = 1.2) for CDS, and /a/ = 13.2 (SD = 1.0), /i/ = 12.9 (SD = 1.2), /u/ = 12.8 (SD = 1.1) for ADS. A small proportion of initially segmented vowels (approximately 3.6%) were excluded due to poor signal quality, overlapping speech, or formant tracking errors that could not be reliably corrected. The final token counts per speaker for all vowels and conditions were sufficient for stable estimation of formant frequencies and VSA, based on prior CDS literature (e.g., Steen & Englund, 2022; Sarvasy et al., 2022). Importantly, token counts were well balanced across CDS and ADS conditions for each vowel, with comparable means and standard deviations (see Section 2.1.2 for child demographic information). This balance minimizes potential biases in the within-subjects comparisons (CDS vs. ADS) and supports the reliability of the statistical analyses.

#### 2.4.4. Manual Formant Correction and Quality Control

Consistent with standard practice in acoustic phonetic research (e.g., Li et al., 2019; Vorperian & Kent, 2007), LPC-generated formant estimates were visually inspected for each token. Visual inspection was performed by overlaying the LPC-derived formant tracks on wideband spectrograms (300 Hz bandwidth). Formants were considered spurious or missing and subject to manual correction if: (a) the LPC trace showed a sudden, physiologically implausible jump in F_1_ or F_2_ (e.g., a change >200 Hz between adjacent time points) that did not correspond to visible spectral energy on the wideband spectrogram; (b) the LPC algorithm failed to track a formant due to weak spectral energy, particularly in high vowels (e.g., /i/ or /u/) or in segments produced with reduced amplitude; or (c) the LPC trace tracked a harmonic or a nasal formant instead of the intended oral formant. In such cases, manual correction was performed by visually matching the LPC trace to the spectral peaks visible on the wideband spectrogram. All manual corrections were performed by a single trained analyst with extensive experience in acoustic phonetic analysis, following a standardized correction protocol.

#### 2.4.5. Reliability of Manual Segmentation and Formant Extraction

Given that vowel onset and offset boundaries were manually identified and that formant values were extracted at the vowel midpoint, we assessed both inter-rater and intra-rater reliability for segmentation and formant measurements. All reliability analyses were conducted on a randomly selected subset of 10% of the total vowel tokens (*n* = 273), stratified by vowel category (/a/, /i/, /u/) and condition (CDS, ADS) to ensure balanced representation.

Inter-rater reliability. A second trained analyst, who was blind to the study hypotheses and condition labels, independently re-segmented all tokens in the subset and re-extracted F_1_ and F_2_ at the vowel midpoint following the identical protocol described above. For vowel duration (i.e., boundary placement accuracy), the intraclass correlation coefficient (ICC, two-way random effects, absolute agreement) between the two raters was 0.94 (95% CI: 0.92–0.96), indicating excellent agreement. The mean absolute difference in duration between raters was 12 ms (SD = 8 ms), which is comparable to values reported in similar CDS studies (e.g., Steen & Englund, 2022; Lee et al., 1999).

For formant extraction, inter-rater reliability was also high. Pearson correlations between raters were r = 0.93 for F_1_ and r = 0.91 for F_2_. ICCs (two-way random, absolute agreement) were 0.94 (95% CI: 0.92–0.96) for F_1_ and 0.92 (95% CI: 0.89–0.94) for F_2_. The mean absolute differences between raters were 42 Hz (SD = 31 Hz) for F_1_ and 58 Hz (SD = 44 Hz) for F_2_. These differences are consistent with typical measurement error in formant analysis (approximately 5–10% of formant frequency values) and are well within the acceptable range reported in the phonetic literature (e.g., Lee et al., 1999; Vorperian & Kent, 2007).

Intra-rater reliability. To assess intra-rater reliability, the primary analyst re-analyzed the same subset of tokens two weeks after the initial analysis, following the identical protocol and without reference to the original measurements. Intra-rater reliability was excellent: ICCs were 0.96 for vowel duration, 0.95 for F_1_, and 0.93 for F_2_. The mean absolute differences between the two sessions were 9 ms (SD = 6 ms) for duration, 38 Hz (SD = 28 Hz) for F_1_, and 51 Hz (SD = 39 Hz) for F_2_.

These reliability estimates confirm that manual segmentation and formant extraction were performed consistently and accurately. All discrepancies between raters were resolved through consensus discussion before finalizing the dataset used for statistical analyses. The high inter-rater and intra-rater reliability support the robustness of the acoustic measurements reported in this study.

#### 2.4.6. Outlier Handling

Extreme outliers in acoustic measures (F_0_, F_0_ range, duration, F_1_, F_2_, and VSA) were identified using the boxplot method, with outliers defined as values greater than 1.5 × the interquartile range (IQR) above the 75th percentile or below the 25th percentile. Outliers were detected in a small number of tokens across all measures: for F_1_, 11 outliers (0.4% of all tokens) were identified; for F_2_, 14 outliers (0.5%); for F_0_, 9 outliers (0.3%); for duration, 16 outliers (0.6%). Rather than excluding these tokens entirely, which could reduce statistical power and introduce bias, we used winsorization: outlier values were replaced with the nearest value within the non-outlier range (i.e., the 75th percentile + 1.5 × IQR for high outliers, or the 25th percentile − 1.5 × IQR for low outliers). This approach reduces the influence of extreme values while retaining all tokens in the analysis (Hampel et al., 1986). Following winsorization, no extreme outliers remained in the dataset. All reported descriptive statistics and inferential analyses are based on the winsorized data.

#### 2.4.7. Derived Measures

Fundamental frequency was extracted using the Dr. Speech software’s autocorrelation-based pitch tracking algorithm, with pitch range set to 75–500 Hz for female speakers, following the system manual recommendations. For each vowel token, F_0_ was sampled at the temporal midpoint (50% of vowel duration) to match the sampling point used for formant extraction, ensuring temporal alignment across acoustic measures. F_0_ range was calculated as the difference between the maximum and minimum F_0_ values within each vowel segment (i.e., between the vowel onset and offset boundaries). This within-segment F_0_ range reflects local pitch variability during vowel production, as opposed to utterance-level pitch range.

Vowel duration was measured directly from the waveform and wideband spectrogram as the time interval (in seconds) between manually marked vowel onset and offset, based on visual inspection of the waveform amplitude envelope and spectrographic cues (e.g., formant onset/offset, changes in spectral energy). Duration was not derived from F_1_, F_2_, or F_0_ values.

F_0_, F_0_ range, F_1_, and F_2_ were measured in hertz (Hz). All values reported are raw, non-normalized formant frequencies and fundamental frequency values.

Vowel space area (VSA) was computed from the mean F_1_ and F_2_ values of the three corner vowels using the triangular area formula (Liu et al., 2003):

VSA = 0.5 × |(F_1_(/a/) × (F_2_(/i/) − F_2_(/u/)) + F_1_(/i/) × (F_2_(/u/) − F_2_(/a/)) + F_1_(/u/) × (F_2_(/a/) − F_2_(/i/))|

This formula calculates the area of the triangle formed by the mean F_1_/F_2_ coordinates of /a/, /i/, and /u/ in the F_1_ × F_2_ plane. A single VSA value was computed per speaker per condition (CDS vs. ADS) based on the mean F_1_ and F_2_ values for each vowel across all tokens within that condition.

To summarize the derivation of each acoustic measure:

F_0_ (mean): Autocorrelation-based pitch tracking at vowel midpoint;
F_0_ range: Max F_0_–Min F_0_ within vowel segment;
Vowel duration: Direct measurement from waveform/spectrogram (onset to offset);
F_1_, F_2_: LPC extraction at vowel midpoint;
VSA: Calculated from mean F_1_/F_2_ using triangular formula.

After extraction, all acoustic parameters were imported into Microsoft Excel for initial organization and outlier detection. Statistical analyses were then conducted in SPSS 27.0 (IBM Corp., Armonk, NY, USA). Results were tabulated and archived for reporting.

## 3. Results

### 3.1. Overview of Statistical Approach

For each acoustic parameter and each vowel, separate one-way repeated-measures ANOVAs were conducted with Speech Type (CDS vs. ADS) as the within-subjects factor. This analytic strategy was chosen for three reasons. First, our primary research questions concern the effect of Speech Type separately for each vowel and each acoustic parameter, and separate ANOVAs provide directly interpretable results for each comparison. Second, this approach aligns with prior CDS studies (e.g., Steen & Englund, 2022; Liu et al., 2003; Sarvasy et al., 2022), facilitating cross-study comparability. Third, the design is balanced and fully within-subjects, making repeated-measures ANOVA a straightforward and appropriate method. We conducted a total of 19 separate ANOVAs: for each of the three corner vowels (/a/, /i/, /u/), we ran ANOVAs for five acoustic parameters (F_0_, F_0_ range, duration, F_1_, F_2_), plus one additional ANOVA for VSA (which is computed at the speaker level across all three vowels per condition, rather than at the token level). To control the family-wise Type I error rate due to multiple comparisons, we applied a Bonferroni correction, setting the adjusted significance threshold at α = 0.05/19 ≈ 0.0026. We report both uncorrected *p*-values (for transparency and comparability with prior literature) and indicate which results remain significant after correction.

All assumptions for repeated-measures ANOVA were checked prior to analysis. Normality of the residuals was assessed using Shapiro–Wilk tests (all *p* > 0.05 after winsorization), and sphericity was not applicable given that the within-subjects factor has only two levels (CDS vs. ADS). Effect sizes are reported as partial eta squared (ηp^2^) for all significant and non-significant results to aid interpretation of the magnitude of effects. 

The token counts were balanced across conditions (CDS vs. ADS) and vowels, with approximately 13 tokens per vowel per condition per speaker (see Section 2.4). This balance ensures that the within-subjects comparisons are not confounded by unequal sample sizes across conditions.

We note that while a multivariate approach (e.g., MANOVA) could have been employed, the separate ANOVA approach was preferred for interpretability given that our hypotheses were formulated separately for each acoustic parameter and each vowel. Furthermore, the within-subjects design and balanced token counts across conditions make the repeated-measures ANOVA appropriate. Mixed-effects models were not employed because each teacher interacted with only one child partner, limiting the ability to model random slopes for child-level variables, and because our primary interest lies in the fixed effect of Speech Type rather than random speaker variability.

We report uncorrected *p*-values in the tables and text for transparency and to facilitate comparability with prior CDS literature (e.g., Steen & Englund, 2022; Liu et al., 2003). However, only effects with *p* < 0.0026 are considered statistically significant in our interpretation. Effects with uncorrected *p*-values between 0.0026 and 0.05 are described as “trends” or “numerical increases” that did not survive correction and should be interpreted cautiously.

### 3.2. Fundamental Frequency (F_0_)

As shown in Table 3, mean F_0_ was higher in CDS than in ADS for all three corner vowels (/a/, /i/, and /u/). However, as indicated in Table 4, this difference reached statistical significance only for /u/ when using uncorrected *p*-values (F = 10.862, *p* = 0.002); however, this effect did not survive Bonferroni correction (corrected threshold α = 0.0026) and should therefore be interpreted as a trend. The increases for /a/ and /i/ did not reach significance even at the uncorrected level (*p* > 0.05). This pattern suggests that the pitch-raising effect in teachers’ CDS may be vowel-specific rather than uniform across all vowel categories. The effect size for /u/ F_0_ was large (ηp^2^ = 0.242), indicating that the observed pitch raising, although reaching significance only at the uncorrected level, represents a substantial effect when present. The effect sizes for /a/ (ηp^2^ = 0.073, medium) and /i/ (ηp^2^ = 0.001, negligible) were consistent with their non-significant *p*-values, following Cohen’s (1988) conventions (ηp^2^ = 0.01, 0.06, and 0.14 for small, medium, and large effects, respectively).

### 3.3. Vowel Pitch Range (F_0_ Range)

As shown in Table 5, F_0_ range—defined as the difference between maximum and minimum F_0_ within each vowel segment—was consistently larger in CDS than in ADS across all three vowels. Statistical comparisons (Table 6) revealed that this increase was significant only for /a/ when using uncorrected *p*-values (F = 9.485, *p* = 0.004); however, this effect did not survive Bonferroni correction (corrected threshold α = 0.0026) and should therefore be interpreted as a trend rather than a statistically significant effect. The increases for /i/ (*p* = 0.058) and /u/ (*p* = 0.103) did not reach significance even at the uncorrected level. The /i/ result (*p* = 0.058) approaches conventional significance and may warrant further investigation in larger samples, but it should not be treated as a statistically supported finding in the present study. The wider pitch range in CDS, particularly for /a/, is consistent with the view that teachers modulate prosodic variability to enhance speech salience and maintain children’s attention. The effect size for /a/ F_0_ range was large (ηp^2^ = 0.218), indicating a substantial increase in pitch variability in CDS for this vowel. The effect sizes for /i/ (ηp^2^ = 0.102) and /u/ (ηp^2^ = 0.076) were medium (Cohen, 1988), suggesting that although not statistically significant, the magnitude of the pitch range increase for these vowels may be of practical interest in larger samples.

### 3.4. Vowel Duration

As shown in Table 7, the duration of all three corner vowels was substantially longer in CDS than in ADS. Statistical analyses (Table 8) confirmed that these differences were highly significant for all three vowels (/a/, /i/, and /u/; *p* < 0.001 for all comparisons). The magnitude of the duration increase ranged from approximately 60% (for /u/) to over 90% (for /a/ and /i/), indicating a robust temporal adaptation in teacher CDS. This lengthening effect is consistent with the slower speech rate and increased temporal clarity that are characteristic of CDS in prior literature (Zangl et al., 2005; Sarvasy et al., 2022). Crucially, all three duration effects showed large effect sizes (ηp^2^ = 0.723, 0.570, and 0.584 for /a/, /i/, and /u/, respectively), indicating that vowel lengthening in CDS is not only statistically robust but also practically substantial. These effect sizes suggest that duration is the most consistent and strongest acoustic adaptation in teachers’ CDS. This pattern may reflect an adaptation in which elongated vowels and other temporal features increase acoustic salience and facilitate language learning in young listeners.

### 3.5. The First Formant (F_1_)

As shown in Table 9, F_1_ values were higher in CDS than in ADS for /a/, while no noticeable differences were observed for /i/ and /u/. However, as indicated in Table 10, none of these differences reached statistical significance (all *p* > 0.05). The numerical increase for /a/ is directionally consistent with greater jaw opening in CDS, but the lack of statistical significance suggests that this articulatory adjustment was not systematically implemented across teachers.

### 3.6. Second Formant (F_2_)

As shown in Table 11, F_2_ values were higher in CDS than in ADS for all three vowels. Statistical analysis (Table 12) indicated that this difference was significant for /a/ when using uncorrected *p*-values (F = 7.192, *p* = 0.011); however, this effect did not survive Bonferroni correction (corrected threshold α = 0.0026) and should therefore be interpreted as a trend rather than a statistically significant effect. The increases for /i/ (*p* = 0.059) and /u/ (*p* = 0.128) did not reach significance even at the uncorrected level. Because F_2_ is sensitive to tongue fronting, the trend toward higher F_2_ for /a/ suggests more fronted articulation in CDS, which may enhance the perceptual distinctiveness of this vowel for young listeners. The effect size for /a/ F_2_ was large (ηp^2^ = 0.175), indicating a substantial fronting of /a/ articulation in CDS. The effect sizes for /i/ (ηp^2^ = 0.101) and /u/ (ηp^2^ = 0.067) were medium, suggesting trends that may merit investigation in larger samples.

### 3.7. Vowel Space Area (VSA)

**Figure 1:**
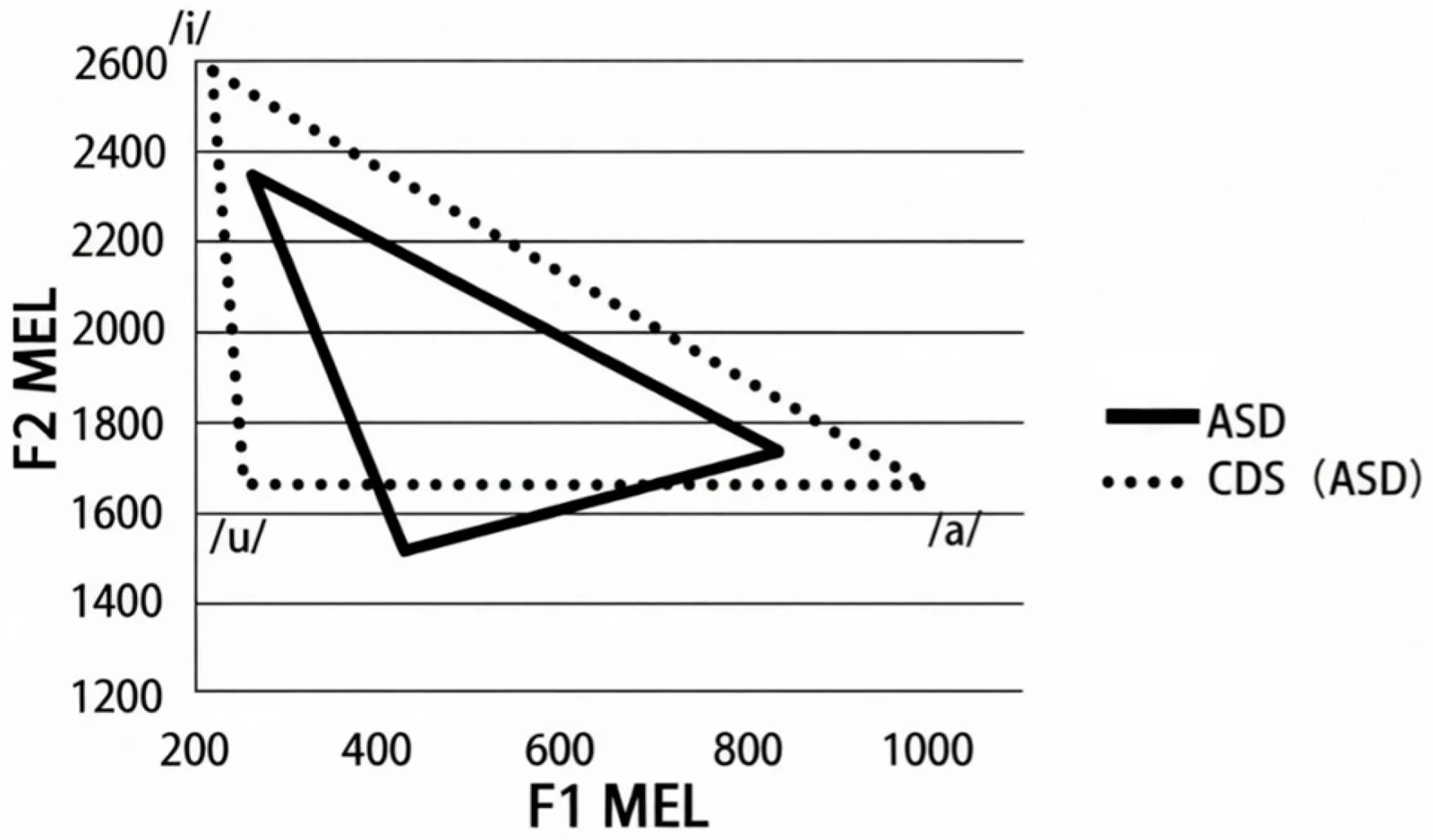
Angular vowel area diagram.

As shown in Table 13, VSA was numerically smaller in CDS than in ADS; however, this difference was not statistically significant (Table 14: F = 0.569, *p* = 0.456, ηp^2^ = 0.016). The triangular vowel space defined by the corner vowels /a/, /i/, and /u/ is illustrated in Figure 1. Thus, the present data do not provide evidence for VSA expansion in teacher CDS. This null finding contrasts with the hyperarticulation hypothesis, which predicts an expanded vowel space in CDS, and is consistent with findings from Norwegian kindergarten teachers (Steen & Englund, 2022). However, as a nonsignificant result, it should be interpreted with caution and does not constitute evidence for VSA reduction. The effect size for VSA was negligible (ηp^2^ = 0.016), consistent with the non-significant *p*-value and indicating that any difference in vowel space area between CDS and ADS is practically minimal.

Note on VSA variability. As shown in Table 13, the standard deviations for VSA are large relative to the means (ADS: SD = 160,215, mean = 160,103; CDS: SD = 182,369, mean = 126,607). This reflects substantial between-teacher variability in vowel space area, which is a well-documented phenomenon in acoustic phonetic research (e.g., Steen & Englund, 2022; McMurray et al., 2013). VSA is computed from F_1_ and F_2_ values (measured in Hz^2^), and individual differences in vocal tract anatomy, dialectal variation, and habitual articulation patterns contribute to large between-speaker differences in baseline vowel space configurations. Importantly, our within-subjects design (each teacher’s CDS compared to her own ADS) controls for this between-speaker variability, making the comparison of CDS vs. ADS within each teacher the appropriate test for detecting speech-style effects. The large standard deviations do not indicate a data error, but rather highlight the importance of the repeated-measures design for isolating the effect of speech style from individual speaker differences.

### 3.8. Exploratory Analyses: The Role of Child Age

Given the age range of the child listeners (3.7–6.6 years) and the interpretive claim that the absence of VSA expansion may be related to children’s developmental stage, we conducted additional exploratory analyses to examine whether teachers’ acoustic adaptations varied as a function of child age. These analyses address the question of whether child age influenced teacher speech production. They were not pre-registered and should be interpreted as hypothesis-generating rather than confirmatory.

First, we treated child age (in years) as a continuous covariate in separate repeated-measures ANCOVAs for each acoustic parameter (F_0_, F_0_ range, duration, F_1_, F_2_, and VSA), with Speech Type (CDS vs. ADS) as the within-subjects factor and child age as the covariate. The main effect of child age was not significant for any acoustic measure (all *p* > 0.05), and the Speech Type × Age interaction was also not significant for any measure (all *p* > 0.05), indicating that the magnitude of CDS–ADS differences did not vary systematically with child age.

Second, we divided the sample into younger (aged 3.7–4.9 years, *n* = 18) and older (aged 5.0–6.6 years, *n* = 17) groups based on a median split (median age = 4.9 years). Separate 2 (Speech Type: CDS, ADS) × 2 (Age Group: younger, older) mixed ANOVAs were conducted for each acoustic parameter. Results revealed no significant main effects of Age Group and no significant Speech Type × Age Group interactions for any of the measures (all *p* > 0.05). For VSA in particular, neither the main effect of Age Group (F(1, 33) = 0.112, *p* = 0.740, ηp^2^ = 0.003) nor the Speech Type × Age Group interaction (F(1, 33) = 0.137, *p* = 0.714, ηp^2^ = 0.004) reached significance.

Taken together, these exploratory analyses did not provide statistical evidence that teachers’ acoustic adaptations—including VSA—varied as a function of child age within the present sample. However, we caution that these null findings may reflect limited statistical power due to the modest sample size (N = 35 teachers, one child per teacher) and the relatively restricted age range. Future studies with larger samples and a wider developmental window (e.g., including infants, toddlers, and school-age children) are needed to robustly test age-related modulation of CDS acoustic features. In light of these results, we have tempered our interpretive claims regarding age-related VSA reduction in the Discussion, treating this possibility as speculative and in need of direct empirical testing.

### 3.9. Supplementary Statistical Details

#### 3.9.1. Effect Sizes and Confidence Intervals

For all repeated-measures ANOVAs, effect sizes are reported as partial eta squared (ηp^2^). Following Cohen’s (1988) conventions for ηp^2^, effect sizes of 0.01, 0.06, and 0.14 are interpreted as small, medium, and large, respectively. 

#### 3.9.2. Assumption Checks

Prior to conducting the repeated-measures ANOVAs, we verified the following assumptions:

Normality of residuals: For each ANOVA model, residuals were computed and tested using Shapiro–Wilk tests. After winsorization of outliers, all Shapiro–Wilk tests were non-significant (all *p* > 0.05), indicating that the normality assumption was satisfied.

Sphericity: Since the within-subjects factor (Speech Type) has only two levels (CDS vs. ADS), sphericity is not applicable (i.e., there is only one pair of repeated measures, so no correction is needed).

Homogeneity of variance: This assumption is not relevant for repeated-measures ANOVA, as each participant serves as their own control.

#### 3.9.3. Confidence Intervals for Significant Effects

For the significant duration effects that survived Bonferroni correction, the 95% confidence intervals for the mean difference (CDS−ADS) were:

/a/ duration: 0.106–0.134 s (*p* < 0.001, ηp^2^ = 0.723);

/i/ duration: 0.096–0.148 s (*p* < 0.001, ηp^2^ = 0.570);

/u/ duration: 0.042–0.074 s (*p* < 0.001, ηp^2^ = 0.584).

These CIs indicate that the duration increases in CDS were substantial and consistently positive across all three vowels.

For the uncorrected significant effects (F_0_ range for /a/, F_2_ for /a/, and F_0_ for /u/), the 95% CIs for the mean differences are reported in the respective subsections above. However, we emphasize that these effects did not survive Bonferroni correction and should be interpreted with caution.

## 4. Discussion

Overview of hypothesis testing: Before discussing each finding in detail, we summarize which hypotheses were supported by our data. H1 (Pitch) received limited support: F_0_ range for /a/ and mean F_0_ for /u/ showed trends toward elevation in CDS (uncorrected *p* = 0.004 and *p* = 0.002, respectively), but these effects did not survive Bonferroni correction for multiple comparisons. The numerical increases for /a/ mean F_0_ and /i/ mean F_0_ did not reach significance even at the uncorrected level. H2 (Duration) was strongly supported: all three vowels (/a/, /i/, /u/) showed significantly longer durations in CDS than in ADS, surviving Bonferroni correction (all *p* < 0.001). H3 (Formants) received limited support: F_2_ for /a/ showed a trend toward higher values in CDS (uncorrected *p* = 0.011) but did not survive Bonferroni correction. F_1_ showed no significant differences for any vowel, and F_2_ increases for /i/ and /u/ did not reach significance even at the uncorrected level. H4 (VSA) was not supported: VSA did not differ significantly between CDS and ADS. H5 (Vowel-specificity) was confirmed, with adaptations varying across vowels (e.g., duration effects were robust for all vowels, whereas pitch and formant effects were more vowel-specific).

### 4.1. Fundamental Frequency (F_0_) and F_0_ Range

Compared with ADS, /u/ showed a trend toward higher F_0_ (uncorrected *p* = 0.002), while /a/ and /i/ showed numerical increases that did not reach significance even at the uncorrected level. It is important to note that the nonsignificant increases for /a/ and /i/ should not be interpreted as evidence of pitch raising; rather, they reflect a trend that was not statistically reliable in this sample. The vowel-specific pattern—with only /u/ reaching significance—suggests that pitch modulation in CDS may be more selective than previously assumed, possibly influenced by the phonetic or lexical properties of specific vowels. These results are consistent with cross-linguistic findings that have frequently reported elevated pitch and greater pitch variability in CDS. Prior research has suggested that such prosodic features may play a role in attracting children’s attention and supporting language processing (Liu et al., 2003; Sarvasy et al., 2022). However, we caution that our study did not directly test the attentional or learning effects of these acoustic features; the observed patterns should therefore be interpreted as descriptive evidence of acoustic modulation rather than as evidence of functional benefits.

Previous studies indicate that caregivers modulate pitch and other prosodic features to aid word segmentation and recognition (Trainor & Desjardins, 2002; Han et al., 2021). Higher mean pitch and a wider pitch range have also been linked to both immediate and longer-term vocabulary growth in young children (Shi et al., 2023). The heightened F_0_ and broader modulation observed here likely reflect teachers’ attempts to maintain engagement and highlight key lexical items, such as toy names, during interactions.

Moreover, an increased pitch range may also serve affective and regulatory functions. Affirmative and emotionally positive utterances often show higher pitch contours (Jungheim et al., 2014). Elevated pitch in CDS may therefore reflect adaptive social-communicative strategies that acknowledge children’s participation and help sustain attention (Burnham et al., 2002).

### 4.2. Vowel Duration 

Our results show that the durations of /a/, /i/, and /u/ are significantly longer in CDS than in ADS. This pattern is consistent with prior work reporting that CDS is slower, with longer syllables and extended pauses, which provides clearer input for language learners (Zangl et al., 2005; Sarvasy et al., 2022).

Prior research has reported that slower speech rate and vowel lengthening in CDS are associated with improved word segmentation and lexical learning in infants and toddlers (Swanson & Leonard, 1994; Song et al., 2010). In the present study, kindergarten teachers produced significantly longer vowels when addressing children, particularly during interactive naming tasks with novel toys. Based on prior literature (e.g., Song et al., 2010; Zangl et al., 2005), it is plausible that such temporal modifications could provide clearer prosodic cues that facilitate word segmentation. However, we emphasize that our study did not directly measure children’s word learning or comprehension, nor did we manipulate teacher speech to assess its effects. Therefore, the functional benefits of these acoustic adaptations remain speculative and require direct testing in future research.

### 4.3. First (F_1_) and Second Formants (F_2_)

Regarding spectral features, /a/ in CDS showed a higher F_1_ than in ADS, consistent with greater jaw opening and oral cavity expansion and in line with the Hyperarticulation Hypothesis (Steen & Englund, 2022). For F_2_, a trend toward higher values was observed for /a/ (uncorrected *p* = 0.011), with similar numerical increases for /i/ and /u/ that did not reach significance. Because F_2_ is sensitive to tongue fronting, higher values suggest more fronted articulation in CDS, a pattern also reported previously (Steen & Englund, 2022).

These formant adjustments are consistent with the possibility that teachers enhance the acoustic salience of vowels, which—based on prior work on hyperarticulation in CDS (Cristia & Seidl, 2014; Liu et al., 2003)—may facilitate phonetic perception. A more fronted tongue position during vowel production may provide clearer acoustic cues for young listeners. However, we did not directly test whether these acoustic differences translate into improved perception or production by children; this remains an empirical question for future research.

### 4.4. Vowel Space Area (VSA)

Contrary to the Hyperarticulation Hypothesis and many previous studies reporting expanded vowel space in CDS (Liu et al., 2009; Cristia & Seidl, 2014; Sarvasy et al., 2022), the current study found a numerically smaller but non-significant VSA in CDS compared to ADS (F = 0.569, *p* = 0.456, ηp^2^ = 0.016). It is important to emphasize that this null finding does not provide evidence for VSA reduction; rather, it indicates that we did not find statistically reliable evidence of VSA expansion. The numerical trend is consistent with findings from Norwegian kindergarten teachers that also showed reduced VSA in CDS addressed to preschoolers (Steen & Englund, 2022), but this pattern should be interpreted with caution given the lack of statistical significance. The absence of a significant effect may reflect true absence of VSA modulation in this age group, insufficient statistical power, or substantial between-teacher variability (as reflected in the large standard deviations).

One possible interpretation of the absence of significant VSA expansion—though speculative and not directly tested by our data—is that it may be related to the age of the child listeners. Many studies that report expanded VSA focus on infants aged 0–3 years (e.g., Liu et al., 2003; Kuhl et al., 1997), whereas the children in the present study were 3–6 years old. At this later developmental stage, children have largely acquired the core vowel system of Mandarin (Hao, 2018; Wong et al., 2017), and teachers may consequently produce less articulatory exaggeration compared to speech directed at infants. However, we emphasize that our exploratory analyses treating child age as a covariate or grouping variable did not yield statistically significant age-related differences in VSA or other acoustic measures. Thus, while the age-based interpretation is theoretically plausible, it remains speculative and was not empirically supported by our data. Alternative explanations—including task constraints (structured elicitation vs. naturalistic free play), speaker characteristics (teachers as professionals vs. parents as primary caregivers), and statistical power (given the sample size of 35 and large between-teacher variability)—cannot be ruled out. Replication studies with larger samples, wider age ranges, and more naturalistic recording conditions are needed before any firm conclusions can be drawn regarding the role of child age in modulating VSA in teacher-directed CDS.

#### 4.4.1. Situating the VSA Finding in the Broader Literature

The absence of VSA expansion in our study joins a growing body of evidence that challenges the universality of vowel space expansion in CDS. While early studies—predominantly with English-learning infants in naturalistic mother–infant interactions—reported robust VSA expansion (e.g., Kuhl et al., 1997; Liu et al., 2003), more recent studies have yielded mixed findings. For example, Steen and Englund (2022) found numerically smaller VSA in Norwegian kindergarten teachers’ CDS compared to ADS (though not statistically significant in all comparisons). Similarly, McMurray et al. (2013) reported that VSA expansion in infant-directed speech is substantially smaller than previously claimed and may not be a universal feature of CDS. Miyazawa et al. (2022) found that VSA expansion varied considerably across caregivers and was not consistently observed even in infant-directed speech.

What explains these discrepancies? We propose three factors. First, listener age likely plays a role. Most studies reporting VSA expansion have focused on infants under 12 months (e.g., Liu et al., 2003; Kuhl et al., 1997), whereas studies with older children (preschoolers or older) tend to find reduced or absent VSA expansion (Steen & Englund, 2022; the present study). This pattern is consistent with developmental tuning accounts (Soderstrom, 2007; Snow, 1995), which propose that speakers tailor their speech to the listener’s developmental level. Second, speaker role (parents vs. teachers) may matter. Teachers, as professionals, may adopt a more controlled, pedagogical register that prioritizes temporal clarity (longer durations) over articulatory hyperarticulation (vowel space expansion), whereas parents interacting with their own infants may produce more affectively modulated, hyperarticulated speech (Steen & Englund, 2022). Third, the tonal nature of the language may interact with VSA patterns. In tonal languages such as Mandarin, lexical tones already provide rich prosodic information, potentially reducing the need for vowel space expansion as an additional cue for phonetic clarity. Cross-linguistic comparisons between tonal and non-tonal languages are needed to test this hypothesis.

Taken together, these considerations suggest that VSA expansion in CDS is not a universal, invariant feature, but rather a context- and age-sensitive modulation that depends on the interplay of listener characteristics, speaker roles, and language-specific phonological structure.

#### 4.4.2. Theoretical Integration: Hyperarticulation vs. Pragmatic Hypotheses

Taking the full pattern of results together, our findings provide mixed support for the hyperarticulation hypothesis. On one hand, the robust duration effects across all three vowels (H2) and the trend toward higher F_2_ for /a/ (H3) are consistent with the hypothesis that teachers enhance phonetic clarity in CDS. On the other hand, the absence of VSA expansion (H4), the lack of significant F_1_ effects for any vowel, and the lack of significant F_2_ effects for /i/ and /u/ directly contradict the strong prediction that speakers globally expand the vowel space in CDS. Furthermore, the vowel-specific pattern of F_0_ and F_0_ range effects suggests that pitch modulation is not uniformly applied across all vowels, which is not clearly predicted by either framework.

These results are more consistent with the Pragmatic/Functional Hypothesis in some respects: the robust duration lengthening and wider pitch range for /a/ may serve to capture children’s attention and facilitate word segmentation (Burnham et al., 2002; Nencheva et al., 2021), without requiring global vowel space expansion. The vowel-specific nature of the effects—particularly the fact that duration was the only measure that showed consistent significant increases across all three vowels—suggests that temporal enhancements may be the primary acoustic strategy teachers use to modulate speech for preschoolers, rather than articulatory hyperarticulation of vowel targets.

Importantly, the age of the child listeners likely plays a moderating role. Studies reporting strong VSA expansion have predominantly focused on infants aged 0–3 years (Liu et al., 2003; Kuhl et al., 1997), when phonological categories are still being established. In contrast, the preschoolers in our study (3–6 years) have largely acquired the core vowel system of Mandarin (Hao, 2018; Wong et al., 2017). Teachers may therefore rely less on vowel hyperarticulation and more on prosodic and temporal enhancements—such as longer durations and wider pitch ranges—as scaffolding mechanisms for this age group. This interpretation is consistent with developmental tuning accounts of CDS (Soderstrom, 2007; Snow, 1995), which posit that speakers adapt different acoustic features to meet the changing communicative needs of the child listener.

In sum, while our findings do not fully support the strong version of the hyperarticulation hypothesis (i.e., global VSA expansion), they are consistent with a weaker, age-sensitive version in which duration and prosodic enhancements serve as primary acoustic adaptations in teacher CDS directed at preschoolers. Direct tests of this interpretation—including comparisons across different age groups and direct measures of children’s perception—are needed in future research.

### 4.5. Implications and Contributions

These findings highlight the adaptive nature of CDS in Mandarin-speaking preschool settings. The robust duration effects, combined with more selective pitch and formant adjustments, suggest that teachers prioritize temporal clarity over global articulatory hyperarticulation when addressing preschoolers. This pattern is consistent with developmental tuning accounts (Soderstrom, 2007; Snow, 1995), which propose that speakers adapt different acoustic features to meet the changing communicative needs of the child listener. The absence of VSA expansion challenges the strong version of the hyperarticulation hypothesis and suggests that CDS adaptations are age- and context-sensitive rather than universal. From an applied perspective, these descriptive findings may inform future hypothesis-driven research on teacher training and classroom communication, though causal links between specific acoustic features and children’s language outcomes remain to be established.

## 5. Conclusions

This study provides empirical evidence on the acoustic features of CDS in Mandarin Chinese within preschool educational contexts. Empirically, our findings demonstrate one robust set of statistically significant differences between CDS and ADS that survived Bonferroni correction for multiple comparisons: teachers produced significantly longer vowel durations for /a/, /i/, and /u/ in CDS (all *p* < 0.001). In addition, we observed descriptive trends—which did not survive Bonferroni correction—toward wider F_0_ range for /a/ (uncorrected *p* = 0.004), higher F_2_ for /a/ (uncorrected *p* = 0.011), and higher mean F_0_ for /u/ (uncorrected *p* = 0.002); these effects are not considered statistically significant in the present study and should be interpreted with caution. VSA did not differ significantly between CDS and ADS.

These acoustic patterns—particularly the robust duration effects observed across all three vowels and the vowel-specific nature of pitch and formant adjustments—provide descriptive evidence that Mandarin-speaking kindergarten teachers systematically modulate their speech when addressing children, especially in the temporal domain. However, the present study was not designed to test whether these acoustic adaptations influence children’s language outcomes; consequently, any interpretations regarding functional or educational benefits remain speculative and hypothesis-generating. Future research employing experimental manipulations or longitudinal designs with direct measures of child outcomes is needed to determine whether, and to what extent, teacher speech adaptations benefit children’s language development.

We did not find statistically significant evidence of VSA expansion—a null finding descriptively consistent with Norwegian preschool studies (Steen & Englund, 2022) but contrasting with VSA expansion reported in infant-directed speech (e.g., Liu et al., 2003). This null finding may reflect true absence of VSA modulation in this age group, insufficient statistical power, or substantial between-teacher variability; replication studies with larger samples are needed before firm conclusions can be drawn.

In sum, this study provides descriptive evidence of acoustic modulation in Mandarin-speaking kindergarten teachers’ child-directed speech. The functional significance of these adaptations for children’s language processing and learning remains to be established through future research.

### Limitations and Future Directions

Several limitations should be acknowledged. First, our sample comprised only female teachers from a single urban region in China, limiting generalizability to male teachers or diverse educational contexts. Second, the study is purely descriptive: we did not measure children’s language outcomes, comprehension, or learning, so any functional or educational implications remain speculative. Third, the structured elicitation protocol, while ensuring cross-participant comparability, may have constrained the naturalness of interactions compared to unscripted classroom discourse. Fourth, our statistical approach using separate ANOVAs with Bonferroni correction, while appropriate for our hypothesis structure, may increase Type II error risk; future studies with larger samples could employ mixed-effects models or multivariate approaches. Fifth, the study was not designed or powered to systematically test child-level moderators (e.g., age, gender, language proficiency), and our exploratory analyses of these factors yielded null results that require replication. Finally, while the corner vowels provide a well-validated index of global vowel space, Mandarin’s richer vowel inventory (including mid vowels, front rounded vowels, and diphthongs) warrants future investigation.

Future research should incorporate more diverse speaker samples, more naturalistic recording conditions, direct measures of child learning outcomes, and larger sample sizes to robustly test whether and how teacher speech adaptations influence preschoolers’ language development. Experimental and longitudinal designs are particularly needed to establish causal links between specific acoustic features and children’s processing or vocabulary growth.

## Figures and Tables

**Table 1:** Descriptive Statistics of Kindergarten Teachers.

	Gender	Age	Years of Teaching Experience	Education Level	First Language
1	Female	36	11	Bachelor	Mandarin
2	Female	31	10	Bachelor	Mandarin
3	Female	29	7	Bachelor	Mandarin
4	Female	24	2	Bachelor	Mandarin
5	Female	33	8	Bachelor	Mandarin
6	Female	30	6	Bachelor	Mandarin
7	Female	26	4	Bachelor	Mandarin
8	Female	26	5	Bachelor	Mandarin
9	Female	39	5	Bachelor	Mandarin
10	Female	43	21	Bachelor	Mandarin
11	Female	35	9	Bachelor	Mandarin
12	Female	32	10	Bachelor	Mandarin
13	Female	30	7	Bachelor	Mandarin
14	Female	30	8	Bachelor	Mandarin
15	Female	26	4	Bachelor	Mandarin
16	Female	31	7	Bachelor	Mandarin
17	Female	36	13	Bachelor	Mandarin
18	Female	26	4	Bachelor	Mandarin
19	Female	27	5	Bachelor	Mandarin
20	Female	30	9	Bachelor	Mandarin
21	Female	26	3	Bachelor	Mandarin
22	Female	30	8	Bachelor	Mandarin
23	Female	31	7	Bachelor	Mandarin
24	Female	34	9	Bachelor	Mandarin
25	Female	26	4	Bachelor	Mandarin
26	Female	30	6	Bachelor	Mandarin
27	Female	24	3	Bachelor	Mandarin
28	Female	33	10	Bachelor	Mandarin
29	Female	23	2	Bachelor	Mandarin
30	Female	25	4	Bachelor	Mandarin
31	Female	28	4	Bachelor	Mandarin
32	Female	49	24	Associate	Mandarin
33	Female	35	4	Associate	Mandarin
34	Female	30	9	Bachelor	Mandarin
35	Female	27	1.5	Bachelor	Mandarin

**Table 2:** Preschool Participants.

ID	Gender	Age (Years)	First Language
1	Male	4.3	Mandarin
2	Male	4.2	Mandarin
3	Male	4.2	Mandarin
4	Male	6.6	Mandarin
5	Male	4.8	Mandarin
6	Male	3.7	Mandarin
7	Male	6.5	Mandarin
8	Male	5.6	Mandarin
9	Male	5.9	Mandarin
10	Female	3.9	Mandarin
11	Male	3.7	Mandarin
12	Male	3.8	Mandarin
13	Male	3.9	Mandarin
14	Female	3.8	Mandarin
15	Female	3.9	Mandarin
16	Female	5.9	Mandarin
17	Female	6	Mandarin
18	Female	4.9	Mandarin
19	Female	3.8	Mandarin
20	Female	3.9	Mandarin
21	Female	3.8	Mandarin
22	Female	4.8	Mandarin
23	Female	4	Mandarin
24	Female	5	Mandarin
25	Female	5	Mandarin
26	Female	6	Mandarin
27	Female	6	Mandarin
28	Female	5.5	Mandarin
29	Female	5.5	Mandarin
30	Female	5	Mandarin
31	Female	5	Mandarin
32	Female	5.5	Mandarin
33	Female	6	Mandarin
34	Female	6.5	Mandarin
35	Female	4	Mandarin

**Table 3:** Descriptive Statistics of Fundamental Frequency (F_0_) for Corner Vowels /a/, /i/, and /u/.

	Speech Type	Mean	SD
/a/	ADS	224.314	27.817
	CDS	236.257	36.829
/i/	ADS	207.735	42.728
	CDS	208.735	35.984
/u/	ADS	200.559	30.179
	CDS	229.647	41.141

Note: Values are presented as mean (SD).

**Table 4:** Main Effects of Speech Type (CDS vs. ADS) on F_0_ for Corner Vowels.

Vowel	Speech Type	df	F	*p*	ηp^2^
/a/	ADS	1	F(1, 34) = 2.661	0.112	0.073
	CDS				
/i/	ADS	1	F(1, 34) = 0.02	0.889	0.001
	CDS				
/u/	ADS	1	F(1, 34) = 10.862	0.002 *	0.242
	CDS				

Note: * *p* < 0.05.

**Table 5:** Descriptive Statistics of F_0_ Range for Corner Vowels in CDS and ADS.

Vowel	Speech Type	Mean	SD
/a/	ADS	886.529	322.969
	CDS	1126.311	435.084
/i/	ADS	1874.957	388.087
	CDS	2114.843	590.489
/u/	ADS	1203.534	602.301
	CDS	1452.329	733.844

Note: Values are presented as mean (SD).

**Table 6:** Main Effects of Speech Type on the F_0_ Range of Corner Vowels.

Vowel	Speech Type	df	F	*p*	ηp^2^
/a/	ADS	1	9.485	0.004 **	0.218
	CDS				
/i/	ADS	1	3.853	0.058	0.102
	CDS				
/u/	ADS	1	2.801	0.103	0.076
	CDS				

Note: ** *p* < 0.01.

**Table 7:** Descriptive Statistics of Vowel Duration for /a/, /i/, and /u/.

Vowel	Speech Type	Mean	SD
/a/	ADS	0.131	0.0447
	CDS	0.251	0.072
/i/	ADS	0.146	0.065
	CDS	0.268	0.101
/u/	ADS	0.096	0.024
	CDS	0.154	0.041

Note: Values are presented as mean (SD).

**Table 8:** Main-Effect Tests of Speech Type on Vowel Duration.

Vowel	Speech Type	df	F	*p*	ηp^2^
/a/	ADS	1	88.646	<0.01 **	0.723
	CDS				
/i/	ADS	1	45.143	<0.01 **	0.570
	CDS				
/u/	ADS	1	47.683	<0.01 **	0.584
	CDS				

Note: ** *p* < 0.01.

**Table 9:** Descriptive Statistics of the first formant (F_1_).

Vowel	Speech Type	Mean	SD
/a/	ADS	576.660	361.556
	CDS	706.597	458.365
/i/	ADS	339.743	77.278
	CDS	334.509	156.038
/u/	ADS	359.120	69.603
	CDS	343.700	65.082

Note: Values are presented as mean (SD).

**Table 10:** Main-Effect Tests for the first formant (F_1_).

Vowel	Speech Type	df	F	*p*	ηp^2^
/a/	ADS	1	2.273	0.141	0.063
	CDS				
/i/	ADS	1	0.034	0.855	0.001
	CDS				
/u/	ADS	1	1.26	0.269	0.036
	CDS				

**Table 11:** Descriptive Statistics of Second Formant (F_2_).

Vowel	Speech Type	Mean	SD
/a/	ADS	1489.954	349.548
	CDS	1765.486	491.675
/i/	ADS	2214.700	370.786
	CDS	2449.363	580.169
/u/	ADS	1562.660	603.284
	CDS	1795.757	758.953

Note: Values are presented as mean (SD).

**Table 12:** Main-Effect Tests for Second Formant (F_2_).

Vowel	Speech Type	df	F	*p*	ηp^2^
/a/	ADS	1	7.192	<0.011 *	0.175
	CDS				
/i/	ADS	1	3.828	0.059	0.101
	CDS				
/u/	ADS	1	2.436	0.128	0.067
	CDS				

Note: * *p* < 0.05.

**Table 13:** Descriptive Statistics of VSA.

Speech Type	Mean	SD
ADS	160,102.990	160,215.131
CDS	126,606.683	182,369.029

Note: Values are presented as mean (SD).

**Table 14:** Main-Effect Test for Vowel Space Area.

Speech Type	df	F	*p*	ηp^2^
ADS	1	0.569	0.456	0.016
CDS				

## Data Availability

The data that support the findings of this study are available on request from the corresponding author, Minmin Yin (yinminmin@hznu.edu.cn). The data are not publicly available due to privacy and ethical restrictions.

## Abbreviations

The following abbreviations are used in this manuscript:

CDS	Child-directed speech
ADS	Adult-directed speech
VSA	Vowel space area
